# Clinical and radiographic outcome of a bioceramic sealer compared to a resin-based sealer: a retrospective study

**DOI:** 10.1038/s41598-025-85411-6

**Published:** 2025-05-14

**Authors:** Hamzah Ahmad Bani-Younes, Karina Abdelaziz Methqal, Ahmad Abdulhamid Madarati, Alaa Daud

**Affiliations:** 1 Hamad Medical Corporation, Doha, Qatar; 2 Private Dental Center (Sijam Dental Clinics), Riyadh, Saudi Arabia; 3 College of Dental Medicine, QU Health, Qatar University, Doha, Qatar

**Keywords:** Endodontics, Root canal obturation, Resin-based root canal sealers, Bioceramic root canal sealers, Clinical outcome, Oral diseases, Biomaterials

## Abstract

Effective sealing of root canal systems is paramount in achieving favorable and enduring prognosis of root canal treatments (RCTs). Root canal sealers play a pivotal role in sealing the canal system. To date, there is a scarcity of clinical research investigating the implications and long-term performance of bioceramic (BC) sealers. This study aims to compare the treatment outcome of resin-based (RB) and BC root canal sealers. Retrospective data comparing clinical outcomes of 248 endodontically treated teeth was performed utilizing dental records and radiographic assessments. Clinical outcome of the RCTs using both types of sealers were measured by percentages of success rates. Chi square statistical test was used to analyze data at 0.01 and level of significance via SPSS software. Success rate of RCTs was not influenced by patient’s age, gender, tooth type and number of visits. There were no significant differences in the success rate amongst treatment types, obturation techniques, quality of restoration and the sealer type. BC and RB sealers revealed comparable clinical and radiographic outcomes with high success rates. The choice between BC and RB sealers should be guided by case-specific factors, including tooth’s anatomical considerations, patient’s dental health status, obturation techniques and clinician’s skills.

## Introduction

The achievement of a good and enduring prognosis of root canal treatments (RCTs) is contingent upon different factors, of which and is the effective seal of the root canal system. Root canal sealers play a pivotal role by fulfilling key functions such as sealing the interface between the dentinal walls and the root filling core material, sealing the accessory canals, if any, sealing voids and irregularities within the root canal system, and entombs residual bacteria. Additionally, sealers act as lubricants during the obturation process^1^.

The ideal characteristics of root canal sealers encompass biocompatibility, superior sealing ability, the capacity to stimulate bone repair, and antimicrobial properties^2^. Furthermore, achieving robust post-setting adhesion of sealers to the intraradicular dentin has been proposed as a paramount advantage^3^.

A notable advancement in this field is the introduction of bioceramic (BC) sealers, which may enhance RCTs’ outcomes^4^. Craveiro and colleagues, highlighted their role in promoting odontoblast differentiation and releasing bioactive substances^5^. Koch and Brave noted their biocompatibility, evidenced by their acceptance by surrounding tissues^6^. Zhang et al., reported that BC sealers exhibit milder cytotoxicity compared to resin-based sealers; the latter has been the gold standard^7^. Asawaworarit and researchers showed the superior adaptation of BC sealers to the root canal wall and their good penetration into the dentinal tubules, particularly in the apical third of root canals^8^. This factor is instrumental in ensuring adequate sealing in root canals obturated with multiple wave condensation techniques.

In addition, Ginebra and colleagues demonstrated that bioceramic materials generate a chemical bond to dentin through the formation of bone apatite-like material upon their setting^9^. Moreover, research showed that BC sealers do not shrink during setting and exhibit outstanding physical properties, including antibacterial effects, which was attributed to their high alkalinity^8^. A study also reported a slight expansion upon setting, making them suitable for the single cone obturation technique^10^. However, it has previously been highlighted that the challenge in removing BC sealers from the root canal during retreatment procedures is a drawback^11^.

It is important to note that the majority of studies on BC sealers have been in-vitro ones, with limited clinical research. This gap in clinical evidence-based endodontics necessitates further investigation to fully understand the practical implications and long-term performance of BC sealers. Therefore, this study aimed at comparing the clinical treatment outcome of resin-based and bioceramic root canal sealers.

The hypothesis of this study was that there would be no significant differences in the success rate between the Bioceramic and Resin-based sealer.

## Methodology

### Ethical considerations

The research protocol of this retrospective study was approved by “ABHATH”, the Medical Research Center (MRC) at Hamad Medical Corporation (HMC), Qatar, No.: MRC-01-23-156. The ethical approval was obtained without the need for patients’ consent. The study was also conducted in accordance with the World Medical Association’s Helsinki Declaration (2013).

### Study design & samples size

Retrospective data review comparing clinical outcome of two different types of root canal sealers was performed. Dental records of 294 endodontically treated teeth available on the “Carestream” software system (Carestream Dental LLC, Atlanta, GA 30339) at the Dental Department, Alkhor Hospital, Hamad Medical Corporation (HMC) in Qatar were reviewed to assess the clinical outcomes of root canal treated teeth accomplished between January 2019 and January 2022. In addition, intraoral periapical radiographs were taken before and after the treatment and during recall visits by a radiographic technician using parallel technique with cone shift depending on the requirement of the case. Image plate size #2 (Planmeca, Hague Dental Supplies, UK) was used to capture the image and scanned using Planmeca ProScanner 2.0 (Planmeca, Hague Dental Supplies, UK). Radiographic images stored on the “Infinitt” dental radiograph viewer system (©2024 INFINITT North America Inc.) at Alkhor Hospital, Hamad Medical Corporation (HMC) were reviewed to assess the clinical outcomes of these teeth.

The sample size was calculated using G*Power software version. 3.1.9.7 for Windows (Heinrich-Heine-Universität Düsseldorf, Düsseldorf, Germany) with a statistical power of 80% from previous research. The resultant sample size was 50 patients in each group, this number was increased to minimum of 100 in each studied group^12–14^.

### Archiving and extraction of clinical data

Clinical data was retrieved from patient records on “Carestream” software system (Carestream Dental LLC, Atlanta, GA 30339). The data included demographic data (e.g., age, gender), tooth type, treatment details (preoperative and postoperative images, type of root canal sealer used), and additional clinical findings, such as vitality, periapical status and obturation techniques). Cases with inadequate documentation were excluded from the study.

To ensure data confidentiality and ethical compliance, all extracted data were anonymized prior to statistical analysis. These data were stored in a password-protected computer accessible only to the principal investigator. This approach ensured that all relevant clinical and radiographic data were archived, preserving the integrity and reproducibility of the study.

### Study’s inclusion criteria

- Mature permanent teeth with fully formed apices, not requiring any surgical intervention.
- Diagnostically acceptable preoperative, postoperative and recall radiographs.
- Radiographs with adequate obturation quality
- The recall was longer than 12 months following obturation.
- Patients with history of controlled/uncontrolled systemic disease.
- The tooth had received good coronal seal in a timely manner following root canal treatment.

### Study’s exclusion criteria

- Teeth with incompletely formed apices, root caries or perforations.
- Teeth with endodontic procedural errors.
- Teeth with severe periodontal bone disease/bone loss, cracks, or with vertical root fractur
- Cases with inadequate documentation

### Treatment procedure

All data related to RCTs were recorded and no intervention with the treatments was required. The RCTs had been performed by two endodontic specialists (with minimum 10 years’ experience) under high standard techniques. All treatment procedures, after administration of local anaesthesia (from access cavity preparation to coronal restorations) were performed under rubber dam isolation^15^. Upon access cavity preparation and locating canal orifices, canal patency was achieved. Glide path preparation was performed by the ProGlider™ rotary glide path files (Dentsply Maillefer, Ballaigues, Switzerland). Working lengths were determined using an electronic apex locator (Root ZX II; J Morita) and were confirmed radiographically. Root canals were prepared using ProTaper Next rotary files (Dentsply Maillefer, Ballaigues, Switzerland). For retreatment cases, the root filling was removed using Protaper re-treatment instrument D1, D2 and D3 respectively (Dentsply Maillefer) at 350 rpm. Apical size preparations were case specific determined by the operator according to the initial apical size. Irrigation of the root canal systems during instrumentation was achieved using minimum of 5 ml sodium hypochlorite (NaOCL) 2.5% solution for each canal during cleaning and shaping using side-vented needle gauge 27.

The final irrigation protocol included 2 ml of 2.5% NaOCl solution for 1 min followed by 2 ml Ethylene Diamide Tetraacetic Acid 17% (EDTA) solution left in the canal for 2 min with endo activation by endo sonic activator (EndoActivator, Dentsply Sirona, USA) for smear layer removal. Followed by final rinse with 2 ml normal saline for each canal. For cases treated in multiple visits, calcium hydroxide (Metapaste, Meta Biomed, Chungcheongbuk-do, Korea) was injected into the canals and intermediate restorative material (IRM) (Dentsply Sirona, USA)  placed for 2 weeks. Calcium hydroxide was removed using the master apical file and irrigation with 5 ml NaOCl for 2 min followed by the final irrigation protocol (as stated above). Finally, canals were dried with paper points.

The sealer type and obturation techniques were case specific and performed according to the operator’s clinical decision. Two types of sealers were available in the endodontic clinics at Alkor Hospital and being used for routine endodontic practice, namely the Adseal™ resin-based sealer (Meta® Biomed, Korea) and the TotalFill® BC Sealer™ (Premixed Bioceramic material, FKG Dentaire Switzerland). For obturations using TotalFill, single cone technique was used. The sealer was injected into the middle third of the canal. Matched Protaper Next gutta percha cone (Dentsply Sirona) was placed in up and down motion for better sealer penetration. Accessory cones added into wide canals and then gutta percha seared off at canal orifice using calamus heated pluggers (Calamus Dual Thermal obturation system, Dentsply Sirona) and vertically compacted using Buchanan head pluggers (Kerr Dental).

For obturations with Adseal, warm vertical compaction was used. Gutta percha was coated with Adseal and inserted into the canal, calamus heated plugger inserted deep into the canal up to 4–5 mm short of the working length and gutta percha was cut and compacted. Back fill of the canal using calamus was performed by thermal injection of gutta percha which vertically compacted using Buchanan head pluggers.

Following obturation, the final restoration was placed, and the patient was referred to a prosthodontic specialist for cuspal coverage where needed. Usually patients were given one year recall appointments to follow up the case.

### Outcome assessment

Clinical and radiographic data were retrieved from patients records on the “Carestream” dental software. The clinical data included presence of any pain, swelling or sinus tract. The existence of any of the aforementioned clinical signs and symptoms was considered an unsuccessful outcome of the treatment. Radiographic assessment was reviewed by two endodontists. To ensure standardization of the preoperative and postoperative periapical status assessment, intra- and inter-examiner reliability was assessed using Kappa statistics. The results of the intra- and inter-examiner reliability showed high reliability, ranging from 0.861 to 1.00 (95% confidence interval ranged from 0.744 to 1.00). The assessments included the development of a new lesion(s), no changes in lesion size, lesion(s) increase in size, no lesion(s) developed, lesion(s) decrease in size and lesion(s) healed. Cases that developed new lesions, the lesion increased in size or no change in size were considered “non healed”. Cases with lesions decreased in size were considered “healing” and cases with no lesion(s) or when lesions disappeared were considered “healed”. Both healing and healed cases were considered successful outcomes, and no further recall appointment was arranged for the purpose of the study.

### Data analyses

Following the application of inclusion/exclusion criteria, clinical and radiographic outcomes of 248 endodontically treated teeth were investigated. The clinical outcome of the RCTs using both types of sealers were measured by percentages of success rates. The Chi square statistical test was used to analyze data at 0.01 level of significance utilizing the SPSS software (IBM® SPSS®, V25, New York, United States).

## Results

### Patients’ demography

Overall, the *female* patients’ proportion (57.3%) was significantly greater than that of *males* (42.7%) (*P *= *0.022*) (Table 1). The patients age groups were significantly different regardless of the range of age’s groups *(P *< *0.001*). Regarding age distribution, the highest proportion of patients were those aged *31–50* years (49.2%), the lowest proportion of patients were those aged *over 50* years (19%), and the proportion of patients aged *up to 30* was (31.9%).

**Table 1:** The values in brackets represent percentage of patients according to gender within each age category.

Patients gender %	Patients age (%) [%]			
	Up to 30	31–50	Over 50	Total
Male [42.7]	38 (35.8) [45.3]	39 (36.8) [31]	29 (27.4) [60]	**106 (100)**
Female [57.3]	41 (28.9) [54.7]	83 (48.5) [69]	18 (12.7) [40]	**142 (100)**
Total [100]	79 (31.9) [100]	122 (49.2) [100]	47 (19) [100]	248 (100)

### The overall success rate versus types of sealers

The overall success rate of RCTs included in this study was 96%; considering that the cases with “*healing in progress”* (19%) had been categorized as ***successful*** cases alongside with those cases of “*healed”* periapical lesions or cases with “*no lesion”* (no lesions developed) in the healthy periapical tissues (80.3%) (Table 2). On the other hand, a case was considered a ““unsuccessful” if a new lesion had developed, the lesion increased in size, or the case developed symptoms. Therefore, and for better statistical analysis of the rest of the variables, successful outcome will include *healing in progress* cases and cases with *healed* or *no lesion in periapical tissues*. Generally, the outcome of RCTs was correlated with the type of sealers, Adseal and TotalFill.

**Table 2:** The values in brackets represent percentage of cases according to the type of sealer.

Type of sealer	Outcome of RCT upon recall (%)			
	Successful outcome		Unsuccessful outcome	Total
	Healed or no lesion	Healing in progress		
AD Seal [44.8]	80 (74.1)	28 (25.2)	3 (2.7)	111 (100)
	108 (97.3)			
Bioceram [55.2]	111 (85.4)	19 (13.9)	7 (5.1)	137 (100)
	130 (94.9)			
Total [100]	191 (80.3)	47 (19)	10 (4)	248 (100)
	238 (96)			

**Fig. 1:**
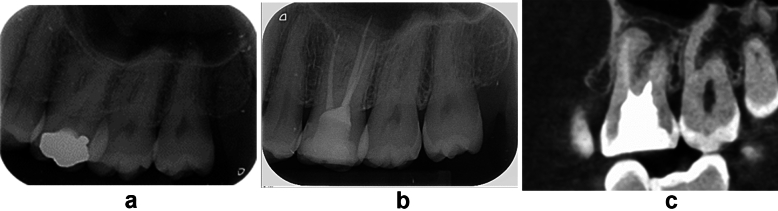
(**a**) Abnormal anatomy found in mesiobuccal root of #26 with a periapical lesion, which was hidden on the radiograph by the Mesiobuccal root, (**b**) tooth obturated (**c**) CBCT scan at one-year recall visit.

An example of a case that was considered *clinically unsuccessful:* a 40-year-old female patient had an RCT performed in a single visit using the BC sealer. On the one-year recall visit, tooth #26 was painful on percussion, with no periapical lesion on the recall radiograph. A Cone beam computed tomography (CBCT) scan was obtained (not for the purpose of this study, but to diagnose the cause of pain). An abnormal anatomy was found in the mesiobuccal root with a periapical lesion which was hidden on the radiograph by the buccal roots (Figure 1)

### Impact of patients’ gender and the sealer type on RCTs outcomes

The success rate of RCTs done for *male* patients (95.3%) was not significantly different from that for *female* patients (96.5%) (Table 3). Hence, the patients’ gender did not affect the success rate of RCTs upon recalls *(P *+ *0.883)*. The success rate of *Adseal* and Totalfill within *Male* patients (95.7 and 94.9%, respectively) and with *Females* patients (95.3 and 98.4%) were all not statistically significant (*P *= *0.519*).

**Table 3:** Values in brackets represent percentage of cases according to the patient’s gender and type of sealer.

Patients’ gender	Type of sealer	Outcome of RCT upon recall (%)		
		Successful outcomes	Unsuccessful outcomes	Total
Male [42.7]	Adseal [44.3]	45 (95.7)	2 (4.3)	47 (100)
	TotalFill [55.7]	56 (94.9)	3 (5.1)	59 (100)
	Total [100]	101 (95.3)	5 (4.9)	106 (100)
Females [57.3]	Adseal [45.1]	63 (98.4)	1 (1.6)	64 (100)
	TotalFill [54.9]	74 (94.9)	4 (5.1)	78 (100)
	Total [100]	137 (96.5)	5 (3.5)	142 (100)
Total (100)	Adseal [44.8]	108 (97.3)	3 (2.7)	111 (100)
	TotalFill [55.2]	130 (94.9)	7 (5.1)	137 (100)
	Total [100]	238 (96)	10 (4)	248 (100)

### Impact of patients age and the sealer type on RCT outcomes

The success rate of RCTs was not influenced by patient’s age (*P *> *0.05*). The success rate of cases for patients aged *up to 30 years* (96.2%) was not significantly different from those of patients aged *31–50* or *over 50* (95.1 and 97.9%, respectively) *(P *= *0.680)*. In fact, there were no significant impact of patients age on success rate of RCTs upon recall regardless the range of patients age (*P *> *0.05* in all cases). The success rate of *Adseal* and *TotalFill* within patients group aged up to 30 Years (96.4 and 96.1%, respectively) were not significantly different from those reported within patients aged 31–50 years (97 and 92.9%, respectively) and patients aged over 50 (100 and 96.7%, respectively) *[P *= *0.526]*, as shown in table 4.

**Table 4:** The values in brackets represent percentage of cases according to the patients’ ages and types of sealers.

Patients’ age	Type of sealer	Outcome of RCT upon recall (%)		
		Successful outcome	Unsuccessful outcome	Total
Up to 30 years [31.9]	Adseal [35.4]	27 (96.4)	1 (3.6)	28 (100)
	TotalFill [64.6]	49 (96.1)	2 (3.9)	51 (100)
	Total [100]	76 (96.2)	3 (3.8)	79 (100)
31–50 years [49.2]	Adseal [54.1]	64 (97)	2 (3)	66 (100)
	TotalFill[45.9]	52 (92.9)	4 (7.1)	56 (100)
	Total [100]	116 (95.1)	6 (4.9)	122 (100)
Over 50 years [19]	Adseal [36.2]	17 (100)	0 (0)	17 (100)
	TotalFill [63.8]	29 (96.7)	1 (3.3)	30 (100)
	Total [100]	46 (97.9)	1 (2.1)	47 (100)
Total [100]		238 (96)	10 (4)	248 (100)

### Impact of tooth type and the sealer type on RCTs outcomes

There were only 2 cases (0.8%) of third molars (maxillary) and 3 cases (1.2%) of lower incisors, hence they were included within upper first and second molars and upper incisors, respectively. Moreover, for better statistical analysis, the 11 canine cases and the incisors were all categorised as anterior teeth. Overall, there was no significant difference between the proportion of *maxillary* and *mandibular* teeth (55.2 and 44.8%, respectively) (*P* = *0.099*) (Table 3). However, while most of the teeth were *molars* (59.7%), the *anterior teeth* composed the lowest proportion (13.7%) *[P *< *0.001].*

There was no significant difference between the maxillary and mandibular teeth regarding the type of sealer used (*P*=*0.701)* (Table 5)*.* However, while there was tendency towards using *Adseal* in *anterior* and *premolar* teeth (52.9 and 57.6%), the significantly higher proportion of *molars* teeth (62.8%) received the *TotalFill* sealer *(P *= *0.013)*.

**Table 5:** The values in brackets represent percentage of cases according to the types of teeth and types of sealers.

Teeth’ types	Type of teeth (%) [%]			
	Anterior	Premolar	Molar	Total
Maxillary (55.2)	27 (19.7)	46 (33.6)	64 (46.7)	137 (100)
Mandibular (44.8)	7 (6.3)	20 (18)	84 (75.7)	111 (100)
Total (100)	34 (13.7)	66 (26.6)	148 (59.7)	248 (100)

Teeth’ types	Type of sealer	Outcome of RCT upon recall (%)		
		Successful outcome	Unsuccessful outcome	Total
Maxillary [55.2]	Adseal [46]	61 (96.8)	2 (3.2)	63 (100)
	TotalFill [54]	71 (95.9)	3 (4.1)	74 (100)
	Total [100]	132 (96.4)	5 (3.6)	137 (100)
Mandibular [44.8]	Adseal [43.2]	47 (97.9)	1 (2.1)	48 (100)
	TotalFill [56.8]	59 (93.7)	4 (4.5)	63 (100)
	Total [100]	106 (95.5)	5 (6.3)	111 (100)
Total [100]		238 (96)	10 (4)	248 (100)

Teeth’ types	Type of Sealer	Outcome of RCT upon recall (%)		
		Successful outcome	Unsuccessful outcome	Total
Molars [59.7]	Adseal [37]	53 (96.4)	2 (3.6)	55 (100)
	TotalFill [62.8]	87 (93.5)	6 (6.5)	93 (100)
	Total [100]	140 (94.6)	8 (5.4)	148 (100)
Premolars [26.6]	ADdseal [57.6]	38 (100)	0 (0)	38 (100)
	TotalFill [42.4]	27 (96.4)	1 (3.6)	28 (100)
	Total [100]	65 (98.5)	1 (1.5)	66 (100)
Anteriors [13.7]	Adseal [52.9]	17 (94.4)	1 (5.6)	18 (100)
	TotalFill [47.1]	16 (100)	0 (0)	16 (100)
	Total [100]	33 (97.1)	1 (2.9)	34 (100)
Total [100]		238 (96)	10 (4)	248 (100)

There were no significant differences in the success rate among the different types of teeth (*molars, premolars and anteriors*) *[P *= *0.386]* as well as between the *maxillary* and *mandibular* teeth *[P *= *0.489].* In addition, the success rates of *Adseal* and TotalFill sealers within *maxillary* teeth (96.4 and 95.9%, respectively) were not significantly different from those reported within *mandibular* teeth (97.9 and 923.7%, respectively) [*P *= *0.329]*. Similarly, the success rates of the *Adseal* and *TotalFill* sealers in molars teeth (96.4 and 93.5%, respectively), premolars (100 and 96.4%, respectively) and anterior teeth (96.4 and 100%, respectively) were not significantly different *[P *= *0.526].*

### Impact of pulp tissues status, treatment type and the sealer type on RCTs outcomes

**Fig. 2:**
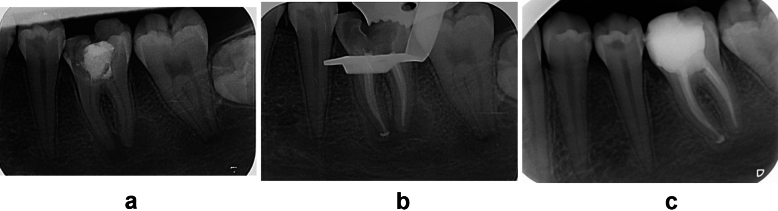
(**a**) Initial periapical radiograph showing periapical lesion. (**b**) Radiograph obtained following obturation and (**c**) Radiograph showing a healing periapical lesion at the 12-month recall.

While most of the cases (60.5%) were *necrotic*, the lowest proportion (10.5%) were *previously treated teeth*; and 29% of cases with *vital pulp tissues* (*P* < *0.001).* Therefore, majority of cases (89.5%) received *primary RCTs* (*P* < *0.001).* However, the success rates of *vital*, *necrotic,* and *previously treated teeth* did not significantly differ (97.2, 94.7 and 100%, respectively) *[P* = *0.360]*. The success rate of cases that received *Adseal* and *TotalFill* sealers within vital pulp tissue (100 and 95.7%, respectively) were not significantly different from those reported with necrotic pulp cases (95.3 and 94.2%, respectively) or previously treated ones (100%) *[P* = *0.519]* as shown in Table 6. There were no significant differences between cases that received *Adseal* and *TotalFill* sealers whether with periapical lesions (96.9 and 94.7%, respectively) or without periapical lesions (97.9 and 96.1%, respectively) *[P* = *0.519]*. An example of a case was a 15-year-old female patient, who required a root canal treatment for tooth #36 with a necrotic pulp and a periapical lesion. RCT took two visits and BC sealer was used. At the 12-month recall, the periapical lesion had healed according to the periapical radiograph obtained, as shown in Fig. 2.

**Table 6:** The values in brackets represent percentage of cases according to the original pulp status, presence/absence of periapical lesions and types of sealers.

Type of RCTs %	Pulp tissues status	Type of sealer	Outcome of RCT upon recall (%)		
			Successful outcome	Unsuccessful outcome	Total
Primary RCTs [89.5]	Vital pulp [29]	Adseal [36.1]	26 (100)	0 (0)	26 (100)
		TotalFill [63.9]	44 (95.7)	2 (4.3)	46 (100)
		Total [100]	70 (97.2)	2 (2.8)	72 (100)
	Necrotic pulp [60.5]	Adseal [42.7]	61 (95.3)	3 (4.7)	64 (100)
		TotalFill [57.3]	81 (94.2)	5 (5.8)	86 (100)
		Total [100]	142 (94.7)	8 (5.3)	150 (100)
Previous RCTs [10.5]		Adseal [80.8]	21 (100)	0 (0)	21 (100)
		TotalFill [19.2]	5 (100)	0 (0)	5 (100)
		Total [100]	26 (100)	0 (0)	26 (100)
Total [100]		238 (96)	10 (4)	248 (100)	Total [100]

Presence of periapical lesion	Type of sealer	Outcome of RCT Upon recall (%)			
		Successful outcome	Unsuccessful outcome	Total	
With apical lesion [48.8]	Adseal [52.9]	62 (96.9)	2 (3.1)	64 (100)	
	TotalFill [47.1]	54 (94.7)	3 (5.3)	57 (100)	
	Total [100]	116 (95.9)	5 (4.1)	121 (100)	
Without apical lesion [51.2]	Adseal [37]	46 (97.9)	1 (2.1)	47 (100)	
	TotalFill [63]	122 (96.1)	5 (3.9)	127 (100)	
	Total [100]	130 (94.9)	7 (5.1)	137 (100)	
Total [100]		238 (96)	10 (4)	248 (100)

### Impact of number of visits and sealer’s types on RCTs outcomes

Six cases needed *3 visits* to complete the RCT, hence for easier statistical analysis and a simplified results presentation, these cases were included with those cases performed in *2 visits* in one category as *multiple* visits. Most of the patients (64.5%) had their RCTs completed in *2 or 3 visits*, while 35.5% of them in a *single session* (*P *< *0.001)* (Table 7). However, the *number of visits* did not affect the success rate after recall periods; as the success rate of a *single-visit* RCTs (96.6%) was statistically similar to that of *multiple-visits* (95.6%) *(P *= *0.235)* (Table 7)*.* Moreover, the success rates of cases that received *Adseal* and *TotalFill* sealers in a *single visit* (96.2 and 96.8%, respectively) were not significantly different from those reported with multiple visits (97.6 and 93.3%, respectively) *[P *= *0.326]*.

**Table 7:** The values in brackets represent percentage of cases according to the number of visits, recalls periods, presence/absence of pain upon recall and types of sealers.

Number of visits %	Type of sealer	Outcome of RCT upon recall (%)		
		Successful outcomes	Unsuccessful outcomes	Total
Single visit [35.5]	Adseal	25 (96.2)	1 (3.1)	26 (100)
	TotalFill	60 (96.8)	2 (3.2)	62 (100)
	Total [100]	85 (96.6)	3 (3.4)	88 (100)
Multiple visits [64.5]	Adseal	83 (97.6)	2 (2.4)	85 (100)
	TotalFill	70 (93.3)	5 (6.7)	75 (100)
	Total [100]	153 (95.6)	7 (4.4)	(100)
Total [100]		238 (96)	10 (4)	248 (100)

Recall period %	Type of sealer	Outcome of RCT upon recall (%)		
		Successful outcomes	Unsuccessful outcomes	Total
Up to 1 year recall [71.4]	Adseal [40.7]	70 (97.2)	2 (2.8)	72 (100)
	TotalFill [59.3]	99 (94.3)	6 (5.7)	105 (100)
	Total [100]	169 (95.5)	8 (4.5)	177 (100)
More than 1 year recall [28.6]	Adseal [54.9]	38 (97.4)	1 (2.6)	39 (100)
	TotalFill [45.1]	31 (96.9)	1 (3.1)	32 (100)
	Total [100]	69 (97.2)	2 (2.8)	71 (100)
Total [100]		238 (96)	10 (4)	248 (100)

Presence of pain upon recall %	Type of sealer	Outcomes of RCT upon recall (%)		
		Successful outcome	Unsuccessful outcomes	Total
No pain [96.8]	Adseal [45.4]	106 (97.2)	3 (2.8)	109 (100)
	TotalFill [54.6]	126 (96.2)	5 (3.8)	131 (100)
	Total [100]	232 (96.7)	8 (3.3)	240 (100)
With pain [3.2]	Adseal [25]	2 (100)	0 (0)	2 (100)
	TotalFILL [75]	4 (66.7)	2 (33.3)	6 (100)
	Total [100]	6 (75)	2 (25)	8 (100)
Total [100]		238 (96)	10 (4)	248 (100)

The proportion of cases observed for *up to 1 year* (71.4%) were significantly greater than that of cases observed for *more than one year* (28.6%) *[P *< *0.001].* Nevertheless, there was no significant difference in the success rate between recall periods (95.5 and 97.2%, respectively) *[P *= *0.729].* Also, the success rates of cases that received *Adseal* and *TotalFill* sealers and observed for up to one year (97.2 and 94.3%, respectively) were not significantly different from those observed for more than one year (97.4 and 96.9%, respectively) *[P *= *0.526]*.

Only 8 cases (3.2%) presented with pain upon recalls; 6 of them were successful (66.7%); with 2 filled with the *Adseal* sealer and 4 were filled with the *TotalFill* one. There was no significant correlation between the success rate and presence/ absence of pain upon recalls *(P* = *0.095).* There were no significant differences in the success rate of cases presented without pain and filled with the *Adseal* (97.2%) and that of cases filled with the *TotalFill* sealer (96.2%) *[P* = *0.519].*

### Impact of obturation technique, quality of restoration and sealers’ type on the RCT outcomes

The majority of cases (85.1%) presented with *sound coronal restorations* upon recalls, which was significantly greater than the proportion of cases that presented with *defected restoration*s (14.9%) *[P *< *0.001]* (Table 8). However, the success rates of both groups (96.7% and 91.9%, respectively) were not significantly different *[P *= *0.361]*. Furthermore, the success rate of cases that received *Adseal* and *TotalFill* sealers with sound coronal restorations (97.7% and 95.7%, respectively) were not significantly different from those presented with defected restorations (93.3% and 90.9%, respectively) *[P *= *0.519]*.

**Table 8:** The values in brackets represent percentage of cases according to the quality of coronal restorations upon recalls, obturation techniques and types of sealers.

Quality of coronal restorations upon recalls %	Type of sealer	Outcomes of RCT upon recall (%)		
		Successful outcome	Unsuccessful outcomes	Total
Sound restorations (85.1)	Adseal [45.5]	94 (97.9)	2 (2.1)	96 (100)
	TotalFill [54.5]	110 (95.7)	5 (4.3)	115 (100)
	Total [100]	204 (96.7)	7 (3.3)	211 (100)
Defected restorations (14.9)	Adseal [40.5]	14 (93.3)	1 (6.7)	15 (100)
	TotalFill [59.5]	20 (90.9)	2 (9.1)	22 (100)
	Total [100]	34 (91.9)	3 (8.1)	37 (100)
Total [100]		238 (96)	10 (4)	248 (100)

Obturation technique %	Type of sealer	Outcomes of RCT upon recall (%)		
		Successful outcomes	Unsuccessful outcomes	Total
Vertical compaction (44)	Adseal	107 (98.2)	2 (1.8)	109 (100)
	TotalFill	0 (0)	0 (0)	0 (100)
	Total [100]	107 (98.2)	2 (0)	109 (100)
Single cone (54.8)	Adseal	0 (0)	0 (0)	0 (100)
	TotalFill	129 (94.9)	7 (5.1)	136 (100)
	Total [100]	129 (94.9)	7 (5.1)	136 (100)
Lateral condensation (1.2)	Adseal	1 (50)	1 (50)	2 (100)
	TotalFill	1 (100)	0 (0)	1(100)
	Total [100]	2 (66.7)	1 (33.3)	3 (100)
Total [100]		238 (96)	10 (4)	248 (100)

Only 3 cases (1.2%) were obturated using the cold lateral condensation (2 using the *Adseal* and 1 using the *TotalFill* sealer). Also, it should be noted that while all cases of vertical compaction techniques were implemented using the *Adseal* sealer, all cases of the single cone technique were implemented using the *TotalFill* Sealer. There was no significant difference between the proportion of cases filled with the *vertical compaction* technique (44%) and that of cases filled with the *single cone* technique (54.8%) *[P *= *0.085]* (Table 8)*.* The combination of obturation techniques and the sealer type did not affect the success rate upon recall as there was no significant difference between the success rate of cases filled with the *vertical compaction* (hence the *Adseal sealer*) (98.2%) and that filled with the *single-cone* technique (the *TotalFill* sealer) (94.9%) *[P *= *0.306]*.

## Discussion

### The overall success rate Vs types of sealers

Bioceramic sealers have garnered significant attention in endodontics due to their advantageous properties, such as biocompatibility, dimensional stability, and their ability to promote hydroxyapatite formation, which are vital for the long-term success of RCTs^16^. A comparative analysis of bioceramic and resin sealers reveals distinctive differences in their clinical outcomes. Studies, such as the one conducted by Al-Haddad et al., indicate that bioceramic sealers exhibit superior sealing ability, enhanced by their expansion upon setting and their capacity to form chemical bonds with dentin^17^. By contrast, while resin-based sealers provide good adhesion, they are prone to shrinkage and have raised concerns regarding their potential cytotoxicity^7^. Moreover, bioceramic sealers demonstrate a high pH during setting, which contributes to their antimicrobial efficacy, a property less pronounced in resin-based sealers^18^. Despite these differences, both types of sealers have shown high clinical success rates of RCTs^19^. Research also underscored the importance of the clinician’s expertise and case selection in determining the clinical outcome^19^ This is in-line with findings from our current study, revealing the high overall success rate (96%) for both types of sealers, with the majority of cases being categorized as either “healed”, “healing in progress” or “no lesion developed”.

### Impact of patients age, gender, and type of sealer on RCT outcome

The age and gender of patients and the type of endodontic sealer used are critical factors influencing the outcome of RCTs^20^. Age-related changes in the dentin, such as decreased cellular content and increased sclerotic changes, can affect the adherence and sealing properties of endodontic sealers, potentially impacting the success of RCT. In the context of sealer type, bioceramic and resin-based sealers exhibit distinct characteristics that interact differently with these age-related dental changes. Bioceramic sealers are known for their excellent biocompatibility and the ability to form hydroxyapatite, which can be advantageous in older patients with more brittle or sclerotic dentin. For instance, Prüllage et al., (2016) has demonstrated the efficient sealing ability and biocompatibility of bioceramic sealers, which can enhance the healing process in the aging dental structure^21^. In contrast, resin-based sealers, with their superior adhesion capabilities as shown by Al-Haddad and colleagues, may be more beneficial in younger patients where the dentin is less sclerotic and can better support resin infiltration^17^. The results of the current clinical study showed no significant impact of patients age on the success rate, including both types of sealers. This highlights the fact that while the sealer type plays a pivotal role, the overall success of RCTs is multifactorial, where the patient’s age and dental health, the clinician’s skill, and the tooth’s anatomical complexities collectively determine the outcome^20^.

### Impact of teeth type and type of sealer on RCT outcome

The efficacy of RCTs is usually significantly influenced by both the type of tooth being treated and the choice of endodontic sealer^22^. The anatomical complexities of different teeth, such as the narrower and more curved canals in premolars and molars compared to anterior teeth, necessitate careful considerations in sealer selection to ensure optimal outcomes^23^. Bioceramic sealers, renowned for their biocompatibility and ability to promote hydroxyapatite formation, are advantageous in complex cases like multi-rooted molars where their flowability and chemical bond formation with dentin are beneficial. Research highlights the efficacy of bioceramic sealers in ensuring a hermetic seal in anatomically challenging teeth^24^. Conversely, resin-based sealers are well known for their superior adhesive properties^25^, which may offer advantages in anterior teeth where the straighter canal anatomy allows for better sealer penetration and adhesion. In the current study, there was no significant difference amongst different teeth nor between maxillary and mandibular teeth in terms of the success rate. This could be owing to the tendency towards using *AD Seal* in anterior and premolar teeth, while molar teeth received Bioceram sealers, demonstrating that the choice between bioceramic and resin sealers for RCT should be tailored based on the specific tooth anatomy. However, further research study to investigate this aspect for longer follow up periods is paramount.

### Impact of pulp tissue status, treatment type and type of sealer on RCT outcome

The outcome of RCT is intricately linked to the status of the pulp tissue, the type of treatment performed, and the choice of endodontic sealer. Pulp tissue status, whether it’s vital, inflamed, or necrotic, can significantly influence the treatment approach and the sealer’s efficacy. For instance, in cases of necrotic pulp or retreatment, where the risk of bacterial contamination is higher, bioceramic sealers, due to their superior biocompatibility and bioactivity, might offer an edge. Studies demonstrated the effectiveness of bioceramic sealers in promoting periapical healing due to their hydroxyapatite-forming ability and high pH, which are advantageous in infected canals^26,27^. On the other hand, resin-based sealers, which are recognized for their excellent sealing ability and adhesion to dentin^28^, might be more suitable for vital pulp cases where a tight seal is crucial to prevent reinfection. The choice between bioceramic and resin sealers can also be influenced by the type of treatment; primary RCTs or retreatments. While bioceramic sealers might be preferred in retreatment cases due to their antimicrobial properties and ability to fill irregular spaces, resin sealers could be more advantageous in primary RCT due to their superior adhesive characteristics. In this study, the overall success rates of *vital*, *necrotic,* and *previously treated teeth* did not significantly differ (97.2, 94.7 and 100%, respectively. This was also not influenced by the type of sealer. Considering that the proportion of retreatment cases was the lowest (10.5%), and that only 29% of cases had vital pulp tissues, generalisation of outcome might prove challenging.

### Impact of number of visits and type of sealer on RCT outcome

The number of visits (single vs multiple) and the type of sealer used (bioceramic vs resin) may influence the long-term outcomes of RCTs, though the differences may not always be significant. A recent study conducted by Coşar et al., on asymptomatic mandibular molar teeth, compared the effect of MTA-based bioceramic (MTA Fillapex) and resin-based (AH Plus) sealers on RCTs’ outcomes in single-visit treatments^29^. The study found no significant differences in treatment outcomes or post-obturation pain intensity between the two groups after a two-year follow-up. This suggests that both MTA Fillapex and AH Plus sealers can be effective in single-visit RCTs performed on asymptomatic mandibular molars with irreversible pulpitis. This coincides with the findings of the current study, whereby the success rate of cases that received either AD-sealers or bioceramic sealers in a single visit treatment were not significantly different from those reported with multiple visits treatments.

### Impact of obturation technique & quality of restoration and type of sealer on RCT outcome

The obturation technique and the quality of the subsequent restoration are pivotal in determining the success of RCTs^30–32^***.*** These factors have been reported to interact with the type of sealer used; bioceramic or resin-based^33^. The obturation technique, whether it is lateral compaction, vertical compaction, or single-cone techniques, plays a crucial role in ensuring the three-dimensional sealing of the root canal system^34^. Bioceramic sealers, are well known for their hydrophilic nature and ability to form a chemical bond with the dentinal walls, may provide a better seal when implementing obturation techniques that allow their flowability and adaptation within the root canal system, such as the single-cone technique. On the other hand, resin-based sealers, characterized by their excellent adhesion and dimensional stability, could be more suitable for other techniques such as the warm vertical compaction, which can better exploit their thermoplastic properties^33^. Similarly in the current study, all cases that were filled by the vertical compaction obturation were performed using the *AD Seal* sealer, while all cases of the single-cone technique were conducted using the bioceramic Sealer. Hence, this could explain why no significant difference in the success rate was identified between cases obturated with the *vertical compaction* (*AD Seal*) (98.2%) and that filled with the *single-cone* technique (*Bioceram* sealer) (94.9%) [P = 0.306].

The quality of the final restoration post-RCT is equally crucial ^31,32,35^*.* A well-sealed coronal restoration that prevents bacterial re-entry is a vital factor regardless of the sealer type used. However, the interaction between the sealer and the restorative material can vary; for instance, bioceramic sealers might be less reactive to moisture and therefore more forgiving in cases where the quality of coronal seal is challenged, compared to resin-based sealers that require a more controlled environment for optimal performance^36^. The majority of cases (85.1%) in the current study presented with *sound coronal restorations* upon recalls. It should be noted that most cases were restored with composite resin, as amalgam is no longer used in the State of Qatar^37^. The success rate of cases that received *ADSeal* and *Totalfill* sealers with or without sound coronal restorations were not significantly different from those presented with defected restorations.

Limitation of the study include the use of conventional periapical radiographs which is two dimensional where some pathological condition or anatomical configurations maybe hidden (Fig. 1). One of the advantages when assessing radiographs in the current study was the fact that the same technician obtained all radiographs for the 248 cases, ensuring technique standardization. Nevertheless, the angle of the radiograph might vary depending on each case. The recall period was at least one year where new lesions can possibly develop if the recall period was extended. Some may argue that the one-year follow up of this study is a relatively short time period. However, this study can be considered a reference for further clinical studies that include longer recall periods, which we believe is paramount.

## Conclusion

Based on the findings of the current clinical study, bioceramic and resin-based sealers revealed comparable clinical and radiographic outcomes with high success rates. The choice between bioceramic and resin sealers should be guided by case-specific factors, including the tooth’s anatomical considerations, the patient’s dental health status, the obturation techniques and the clinician’s skills.

## Data Availability

The data sets generated during and/or analyzed during the current study are available from the corresponding author on reasonable request.
